# α-Actinin1 promotes tumorigenesis and epithelial-mesenchymal transition of gastric cancer via the AKT/GSK3β/β-Catenin pathway

**DOI:** 10.1080/21655979.2021.1967713

**Published:** 2021-09-21

**Authors:** Siwen Zhang, Junfu Wang, Ting Chen, Jiancheng Wang, Ye Wang, Zhu Yu, Kun Zhao, Kaitian Zheng, Yeyang Chen, Zhen Wang, Bopei Li, Congjun Wang, Weijia Huang, Zhao Fu, Junqiang Chen

**Affiliations:** a Department of Gastrointestinal Surgery, The First Affiliated Hospital of Guangxi Medical University, Nanning, China; b Graduate College, The Guangxi Medical University, Guangxi Zhuang Autonomous Region, Nanning, People’s Republic of China

**Keywords:** Gastric cancer, α-Actinin1, epithelial-to-mesenchymal transition, AKT/GSK3Β/β-catenin, apoptosis

## Abstract

α-Actinin1 (ACTN1), an actin cross-linking protein, is implicated in cytokinesis, cell adhesion, and cell migration. In addition, it is involved in the tumorigenesis and development of certain cancers, such as breast cancer. We explored the function of ACTN1 in gastric cancer (GC), which has largely remained unclear. High-throughput sequencing and public microarray datasets from the Gene Expression Omnibus (GEO) and The Cancer Genome Atlas (TCGA) revealed the upregulation of ACTN1 in gastric cancer with a poor prognosis. These results were further verified by western blotting (WB), Real-Time Quantitative polymerase chain reaction (RT-qPCR), and immunohistochemistry. We constructed loss and gain of function gastric cancer cells, which revealed the effect of ACTN1 over-expression on promoting GC cell proliferation, invasion, migration, and inhibited apoptosis. Mechanistic studies revealed that ACTN1 regulates the epithelial-mesenchymal transition (EMT) and tumorigenesis of gastric cancer via the AKT/GSK3β/β-catenin pathway, confirmed by the inhibitor of AKT MK2206. Altogether, these results demonstrated that ACTN1 could be a promising candidate for gastric cancer treatment.

## Introduction

Gastric cancer (GC) ranks the fifth place among global cancers, which also frequently results in cancer-associated mortality [1]. GC is mostly diagnosed at an advanced stage as no early predictor is available. GC has high distal metastasis (DM), local metastasis and relapse rates, with the 5-year survival rate after surgery being only twenty percent [2]. At present, the diagnosis of GC is mainly based on endoscopic biopsy, and the main treatment methods consist of surgery, chemotherapy, and radiotherapy. Despite the fact that with the prevention and screening programs, such as endoscopy, the incidence of GC and GC-related mortality has constantly been decreasing, more than 1 million new cases are estimated to be diagnosed annually with the increasing aging population, the younger onset of GC, and the lack of sensitive noninvasive screening and diagnostic indicators [3]. Therefore, there is an urgent requirement to elucidate the underlying mechanism of its occurrence and development and finding more sensitive targets to diagnose and treat this disease.

ACTN1 is a member of the α-actinin family that cross-links actin filaments to create a network and anchor actin to the cytomembrane for synthesizing structures including cell-matrix adhesion or intercellular adhesion junction [4]. The α-actinins are composed of four isoforms: ACTN1, ACTN2, ACTN3, and ACTN4 that are implicated in cytokinesis, cell adhesion, and cell migration [5,6]. ACTN1 and ACTN 4 are widely expressed in non-muscle cells, whereas ACTN2 and ACTN3 are mainly expressed in muscle cells. Some recent studies have discovered that non-muscle α-actinins occupy positions in tumor genesis and development, which is mainly supposed by deregulated ACTN4 in many kinds of cancer tissues related to normal tissue counterparts [7–10]. Aberrant expression of ACTN4 has been reported in several carcinomas such as breast, colorectal, ovarian, and pancreatic cancers. Loss and gain of function studies have revealed that ACTN4 promotes tumor oncogenesis and metastasis [11–15]. Moreover, ACTN4 has been revealed to function as a nuclear receptor coactivator in Breast Cancer (BC) [9]. Only a few studies are available on the role of ACTN1 in cancers. Apart from the similar properties of binding actin and expression, ACTN1 exhibits a high level of sequence homology (87% amino-acid identity) with ACTN4 [10]. Therefore, it is logically rational to deduce that ACTN1 could play a role similar to that of ACTN4 in tumor development. ACTN1 has been reported to show up-regulation within BC, which participates in cancer progression by inducing partial EMT [16]. Similarly, the aberrant expression of ACTN1 has been observed in oral, colon, hepatocellular carcinoma, acute myeloid leukemia, and lung adenocarcinoma, contributing to tumor oncogenesis and metastasis by diverse signaling pathways such as Hippo signaling and FAK/Src/JAK2/STAT3 signaling [16–23]. These results indicate that ACTN1 may participate in tumor occurrence and development. However, the function of ACTN1 in GC has not been elucidated.

An increasing number of studies have revealed that EMT transition, where tumor cells lose epithelial characteristics and cell-to-cell adhesion, contributes to invasion and metastasis [24–27]. Consequently, EMT promotes detachment of cells, as evidenced by the up-regulated mesenchymal marker levels (like N-cadherin and Vimentin) whereas the down-regulated epithelial marker levels (like E-cadherin) [25,28,29]. Low expression of E-cadherin results from the transcriptional inhibition of transcriptional factors, including Snail1 (Snail), Snail2 (Slug), TWIST1, TWIST2, and ZEB1 [25,30,31]. Some signal transduction pathways, such as NF-kB, PI3K/AKT, Wnt/β-catenin, Notch, TGF-α and TGF-β, are involved in aberrant EMT in carcinomas [31].

Typically, Wnt/β-catenin signal transduction pathway is demonstrated to regulate both carcinogenesis and EMT [30,32–34]. β-catenin has dual functions due to its different cellular localizations. When anchored to the cell membrane, β-catenin functions as a scaffolding protein that links the actin cytoskeleton to cell-to-cell adhesion junctions, primarily composed of E-cadherin. Within the cell nucleus, β-catenin acts as a transcriptional factor, activating the downstream target genes such as cyclin D1, C-Myc, MMP14, Slug, FN1, C-JUN, L1CAM, and CD44 [30,35]. It thereby contributes to cell growth, survival, renewal, regeneration, and other biological processes that are related to tumorigenesis and EMT [36,37]. The β-catenin destruction complex, consisting of APC, GSK3β, AXIN, CK1, PP2A, and β-trcp, binds to and degrades β-catenin [37]. Free β-catenin in the cytoplasm is subsequently phosphorylated, ubiquitinated, and degraded. In several cancers, β-catenin accumulates and gets translocated to the nucleus because the β-catenin destruction complex is inhibited due to mutations in the complex’s components and inactivation of kinases such as GSK3β [35,38].

AKT, a kind of serine/threonine kinase, plays an important role in the PI3K/AKT signaling cascade [39]. Abnormal phosphorylation of AKT is related to poor malignancy and prognosis [40,41]. In addition, GSK3β gets phosphorylated at Ser9 to inhibit its catalytic activity, inducing β-catenin accumulation as well as nuclear import [42–44].

In this study, we investigated the role of ACTN1 in the proliferation, invasion, migration, apoptosis, and EMT of GC cells, as well as the mechanism of ACTN1 affecting GC. we aimed to elucidate the internal mechanism underlying the progression of GC and uncover a potential therapeutic target of GC.

## Materials and methods

### Patient and sample collection

Cancer and para-cancerous tissues all came from patients with GC undergoing surgery in our hospital. The inclusion criteria of patients were: GC was diagnosed by electronic gastroscopy biopsy, and other systemic tumors were excluded, and no chemotherapy or radiotherapy was received before the surgery. The study was approved by the Bioethics Committee of First Affiliated Hospital of Guangxi Medical University. The tissue samples dissected were immediately frozen, followed by storage under – 80°C until use.

### Cell culture and transfection

We acquired the human GC cell lines (including MKN-28, HGC-27, MKN-45 and AGS) from the Cell Bank of the Chinese Academy of Sciences (Shanghai China). In addition, the normal human gastric epithelial GES-1 cell line was provided by Fuxiang Biotechnology Limited Company (Shanghai, China). All cell lines were cultivated within the humid incubator under 5% CO_2_ and 37°C conditions. GES-1 and AGS cells were cultured in DMEM containing 10% fetal bovine serum (Gibco-BRI, USA) and 1% Penicillin-Streptomycin Liquid (Solarbio, Beijing, China). MKN-28 cells were cultured in Roswell Park Memorial Institute (RPMI) 1640 (Gibco-BRI, USA). Specific small interfering RNAs (siRNA1, No. stB0002519A; siRNA2, No. stB0002519B; siRNA3, No. stB0002519C) and negative control siRNA (siR-NC, No. siN0000001-15) were purchased from Guangzhou Ruibo Biotech (Guangzhou China). ACTN1 overexpression plasmid and GV417 empty vector were synthesized at Jikai Gene Biotechnology Limited Company (Shanghai, China). Lipofectamine 3000 (Thermo Fisher Scientific, USA) was used for transfection in line with specific protocols. After transfection for 48 to 72 h, protein and RNA extraction was performed for subsequent experiments.

### RNA isolation and RT-qPCR

The NucleoZOL RNA Isolation Kit (Gene Company Ltd., Germany) was utilized for extracting total RNA, while PrimeScript RT Kit (Takara Bio, USA) was used for reverse transcription in line with specific protocols. After that, the obtained cDNA was preserved under – 80°C. Using GAPDH as an internal reference, we applied the Applied Biosystems 7500 RT-PCR System (ThermoFisher, USA) to amplify target gene. In the meantime, we adopted the SYBR Green RT-qPCR Kit (Takara Bio, USA) to detect the target gene. The primer sequences for the RT-qPCR are listed in Supplement Table S1.

### Western blotting

The RIPA lysis buffer (Solarbio, Beijing, China) that contained phenylmethylsulfonyl fluoride (PMSF) and a phosphatase inhibitor (Solarbio, Beijing, China) was adopted to isolate total protein. Thereafter, protein content was measured using the BCA Protein Assay Kit (Solarbio, Beijing, China). The protein samples were loaded onto an SDS PAGE gel and separated, followed by transfering on the 0.22-µm PVDF membranes (Merck Millipore Co., Ltd.). After blocking the membranes with 5% skim milk under ambient temperature for 30 min, we added the primary antibody to incubate the blots under 4°C overnight. Then, Tris Buffered Saline Tween (TBST) was used to wash blots for 5 min thrice, followed by 1 h of secondary antibody incubation under ambient temperature. After washing, the ECL kit (Thermo Scientific, USA) was used to detect the target proteins. GAPDH (Glyceraldehyde-3-phosphate dehydrogenase) acts as a loading control, and Lamin B1 acts as a loading control for nucleoprotein. The antibodies used included GAPDH (Proteintech, No. 60,004-1-Ig, 1:8000, Wuhan, China), Lamin B1 (Proteintech, No. 12,987-1-AP, 1:4000, Wuhan, China), Vimentin (Proteintech. No. 10,366-1-AP, 1:5000, Wuhan, China), E-cadherin (Proteintech. No. 60,335-1-Ig, 1:5000, Wuhan, China), Snail (Proteintech, No. 13,099-1-AP, 1:500, Wuhan, China), phospho-AKT (Ser473) (CST, No. 9271S 1:1000, USA), AKT (CST, No. 9272 1:1000, USA), phospho-GSK3β (Ser9) (Abcam, No. ab75814, 1:1000, USA), GSK3β (CST, No. 12,456 1:1000, USA), Anti-beta catenin (CST, No. 8480, 1:10,000, USA), Anti-beta catenin (phospho T41 + S45) (Abcam, No. ab81305, 1:10,000, USA), cyclin D1 (Proteintech, No. 26,939-1-AP, 1:5000, Wuhan, China), anti-rabbit IgG (CST, No. 7074, 1:8000, USA), and anti-mouse IgG (CST, No. 7076, 1:8000, USA). The nucleoprotein was separated using the Nuclear Protein Extraction Kit (Solarbio, Beijing, China) according to the mentioned protocols and was stored at – 80°C.

### Immunohistochemistry

In brief, GC and matched non-carcinoma samples were prepared into paraffin sections, followed by deparaffinization and hydration, and high-pressure and high-temperature antigen recovery was performed in sodium citrate buffer (pH 6.0). Sequentially they were incubated with endogenous peroxidase blocker, followed by 12 h of primary antibody (1:1000) incubation under 4°C. Sections were then rinsed by phosphate buffered saline (PBS), followed by an additional 20 min of washing by enzyme-conjugated goat anti-rabbit/mouse IgG polymer, and PBS rinsing thrice. Then the sections were incubated with DAB staining solution for 5 min. After washing with water, the paraffin sections were soaked in hematoxylin staining solution for 15 s and finally distinguished, rinsed and returned to blue. Senior pathologists observed the results. The number of positive cells was divided into five grades according to the ratio, and the staining intensity was divided into four levels. The score was obtained by multiplying both the scores. A score of >4 was suggested to be positive.

### Cell proliferation assay

The EDU incorporation was used to detect cell proliferation. Briefly, the thymus analog (EDU) was added to AGS and MKN28 medium for 3 and 4 h of incubation, separately. Thereafter, cells were subjected to fixation, permeabilization, and nuclear dye DAPI staining according to the instructions mentioned in the BeyoClick™ EdU Cell Proliferation Kit with Alexa Fluor 488 (Beyotime, No. C0071S, Jiangsu, China). The nuclei of all cells were stained blue, whereas the proliferating cells were stained green, which were observed using an inverted fluorescence microscope (magnification, x100; Olympus Corporation). The results were analyzed using ImageJ (EDU positive rate = total area of green light/total area of blue light). All experiments were repeated at least thrice.

### Immunofluorescence assay

Briefly, 4% paraformaldehyde was used to fix cells for 10 min, followed by 10 min of 0.1% Triton-X100 permeabilization. After 30 min of blocking with 10% bovine serum albumin (BSA) under 37°C, the primary antibody against E-cadherin (Proteintech. No. 60,335-1-Ig, 1:200, Wuhan, China), Vimentin (Proteintech. No. 10,366-1-AP, 1:5000, Wuhan, China) or β-catenin (CST, No. 8480, 1:200, USA) was used to incubate cells under 4°C overnight, followed by additional 1 h of incubation by the fluorescent secondary antibody in the dark. The cells were observed using the Leica confocal microscope TCS SP5 (63× objective). Equal laser intensity of each channel was maintained for the different experimental groups. E-cadherin showed red fluorescence using the Alexa Fluor 647-labeled Goat Anti-Mouse IgG (H + L) (Beyotime, No. A0428, 1:50, Shanghai, China) as the secondary fluorescent antibody. Vimentin and β-catenin were stained green with the Alexa Fluor 488-labeled Goat Anti-Rabbit IgG (H + L) (Beyotime, No. A0468, 1:50, Shanghai, China).

### Wound-healing assay

For the wound-healing assay, the cells were seeded in six-well plates and grown close to 90% confluency or more. A sterile pipette tip (200-µL) was used to scratch the monolayer cell plane longitudinally to create a wound. After washing with PBS, cell debris was removed and cells were cultivated in a medium containing 1% FBS. Finally, we obtained images at 0, 24, and 48 h, using an inverted microscope (magnification: 4x; Olympus Corporation). We evaluated the cell migration capacity according to the scratch area measured in the ImageJ software. All experiments were repeated at least thrice.

### Transwell assay

The upper chamber covered with or without Matrigel was added with cell suspension containing 2% FBS and 2 × 10^4^ cells. Next, the lower chamber was added with 800 mL of a medium containing 5% FBS. Thereafter, we further incubated cells under 5% CO_2_ and 37°C conditions for 24 h. Later, 95% ethanol was used to fix cells, followed by 0.1% crystal violet staining. Then, a cotton swab was used to wipe cells in the chamber that had not passed through the membrane. The chambers were observed under an inverted fluorescence microscope (magnification: 100x; Olympus Corporation). We evaluated cell migration and invasion capacities according to the number of membrane-penetrating cells.

### Apoptosis

Cell apoptosis was analyzed through flow cytometry. In brief, after washing by pre-chilled PBS, cells were digested using the trypsin digestion solution without EDTA (Solarbio, Shanghai, China). Later, cells were harvested after centrifugation at 1000 rpm for 5 min, followed by 15 min of 7-AAD (BD Biosciences, No. 559,925, USA) and Annexin-APC staining (BD Biosciences, No. 561,012, USA) in the dark. The results were analyzed with the BD FACTNSAria III flow cytometer (BD Biosciences, USA). Cell apoptosis rate was calculated with the software FlowJo-V10 (BD Biosciences, USA) .

### Public database and bioinformatic tools

We obtained GSE26942 (GC tumors: 205, normal samples: 12), GSE54129 (GC tumors: 111, normal samples: 21), GSE56807 (GC tumors: 5, normal samples: 5), GSE118916 (GC tumors: 15, normal samples: 15) and GSE79973 (GC tumors: 10, normal samples: 10) datasets from GEO database (https://www.ncbi.nlm.nih.gov/geo/) [45–48]. The ACTN1 expression differences between GC tissues and normal tissues in these datasets were compared by Student’s t-test, which were visualized through GraphPad Prism7 (GraphPad Software, Inc, USA). P < 0.05 was regarded as statistically significant. GSE84437 (GC tumors: 433) and GSE62254 (GC tumors: 300) datasets with corresponding clinical and survival information were obtained to uncover the associations between ACTN1 expression and clinicopathologic parameters, which including age, gender, stage, and the TNM classifications of GC patients [49,50]. Sufficient and explicit clinical information was the inclusion criteria. The Kaplan-Meier analysis was performed to evaluate the association between ACTN1 and overall survival (OS) using a log-rank test based on both two datasets. A Chi-square test was used to measure the association between ACTN1 expression and clinical features of GC patients. P < 0.05 was regarded as statistical significance. The GC datasets of TCGA and GTEx were directly analyzed via the GEPIA (http://gepia.cancer-pku.cn/about.html) [51]. The Kaplan–Meier analysis was performed through the Kaplan–Meier Plotter (http://kmplot.com) [52]. The Encyclopedia of RNA Interactomes ENCORI (http://starbase.sysu.edu.cn/index.php) was utilized to detect associations of molecules [53].

## Statistical analysis

All statistical analyses were done though GraphPad Prism7 (GraphPad Software, Inc, USA). Data were presented as means±SD (Standard Deviation) from 3 independent assays. Differences were compared among several groups through one-way ANOVA, whereas those between 2 groups were compared by Student’s t-test. The associations between ACTN1 expression and the clinicopathological features of the patients were analyzed using the Chi-squared test. A difference of P < 0.05 suggested statistical significance.

## Results

We speculated that ACTN1 played the role of an oncogene and facilitated the progression of GC. First, we verified the differential expression of ACTN1 in tumors and adjacent tissues by high-throughput sequencing, using public databases, as well as, assessing the mRNA and protein levels of GC cell lines. Then phenotypic experiments were performed, which demonstrated that ACTN1 promoted the tumorigenesis and EMT of GC. To determine the mechanism, we used WB, RT-qPCR, and immunofluorescence to verify the expression changes of key signaling molecules. The rescue experiment confirmed the results.

### Overexpression of ACTN1 in GC and its relation with poor prognosis

**Figure 1.**
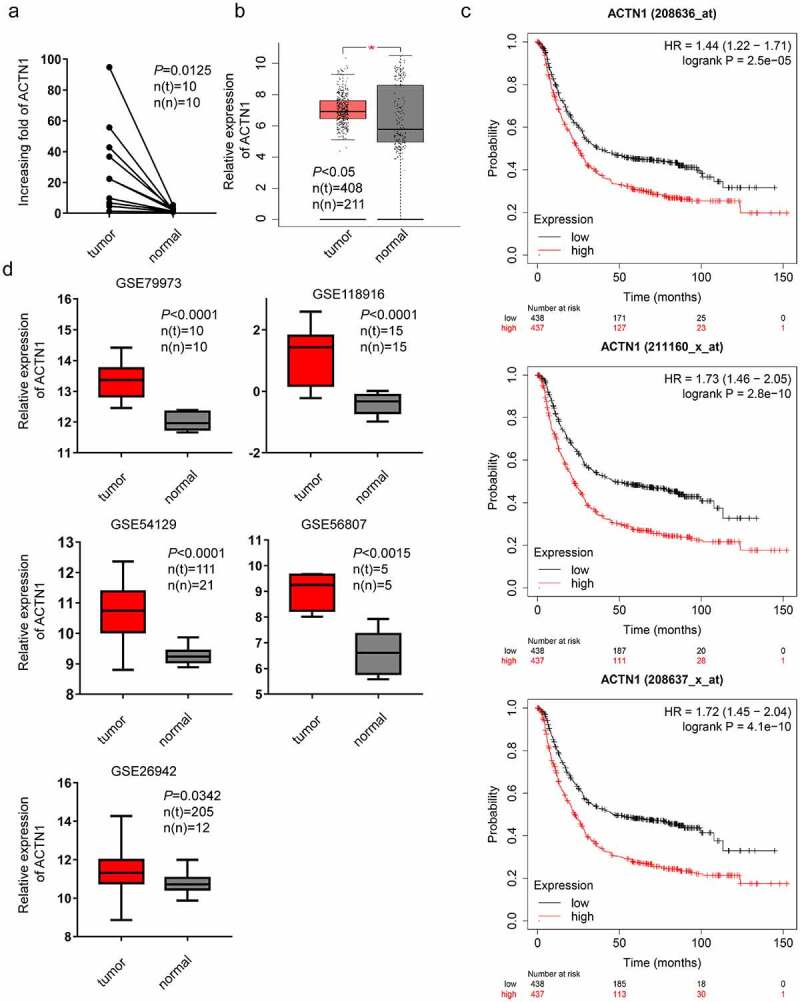
**Aberrant expression of ACTN1 in GC. a** High-throughput sequencing results obtained from 10 pairs of GC tumors (tumor) and adjacent normal tumors (normal). **b** Aberrant expression of ACTN1 between 408 GC samples and 211 non-carcinoma samples obtained from TCGA database and GTEx as per the GEPIA (P < 0.05 *). **c** Multiple gene symbols (208636_at; 208637_x_at; 211160_x_at) showed that ACTN1 was related to poor overall survival as per the Kaplan–Meier Plotter (Log-rank P < 0.001). **d** GSE79973 (tumor n(t) = 10; normal n(n) = 10), GSE118916 (tumor n(t) = 15; normal n(n) = 15), GSE54129 (tumor n(t) = 111; normal n(n) = 21), GSE56807 (tumor n(t) = 5; normal n(n) = 5), GSE26942 (tumor n(t) = 205; normal n(n) = 12)) showing ACTN1 was significantly upregulated in gastric cancer (GC) tumors (P < 0.05 was regarded as statistical significance)

The expression difference between tumor tissues and tumor-adjacent tissues can provide clues to uncover the mechanism of tumorigenesis and development. To understand the underlying mechanisms of GC tumorigenesis, mRNA was isolated from 10 pairs of GC tissues and para-carcinoma tissues for high-throughput sequencing. We found that ACTN1 was overexpressed in GC (P < 0.0125) (Figure 1a). Furthermore, ACTN1 was upregulated in 408 GC samples relative to 211 non-carcinoma adjacent samples according to the public databases TCGA and GTEx as per the GEPIA (P < 0.05) (Figure 1b). According to bioinformatics analysis using the Kaplan–Meier Plotter, ACTN1 over-expression predicted dismal GC prognostic outcome (P < 0.001) (Figure 1c). Similarly, a comparative analysis was conducted between open GEO database (GSE118916, GSE79973, GSE26942, GSE56807, and GSE54129) [45–48] in GC samples and non-carcinoma adjacent samples, revealing that ACTN1 was overexpressed in GC (P < 0.05) (Figure 1d). The range of the expression levels of ACTN1 varied a lot among these datasets, which might be caused by different platforms and probes used. Furthermore, we determined the association between ACTN1 and the clinical-pathological parameters of GC patients based on the public GEO datasets (GES62254, GES84437) [49,50]. The results showed that ACTN1 up-regulation was significantly associated with advanced Stage (III+IV) (P = 0.002) and T classification (T3+ T4) (P = 0.018) in GSE62254 (Table 1, Supplement Fig. S2a). The strong association with T classification (P = 0.002) was similarly confirmed in GSE84437 (Table 2, Supplement Fig. S2b). The survival analysis showed that the high-ACTN1 groups were associated with decreased survival (GES62254: P < 0.001; GES84437: P < 0.002).

**Table 1.** Correlation between ACTN1 expression and clinicopathologic characteristics of gastric cancer patients (GSE62254)

N	Overall	low	high	*p*
	298	171	127	
**Age(mean(SD))**	61.89(11.38)	62.27(11.37)	61.38(11.41)	0.505
**Gender(%)**				0.27
Female	101(33.9)	53(31.0)	48(37.8)	
Male	197(66.1)	118(69.0)	79(62.2)	
**Stage(%)**				**0.002**
I+ II	126(42.3)	86(50.3)	40(31.5)	
III+IV	172(57.7)	85(49.7)	87(68.5)	
**T(%)**				**0.018**
T1+ T2	186(62.4)	117(68.4)	69(54.3)	
T3+ T4	112(37.6)	54(31.6)	58(45.7)	
**M(%)**				0.103
M0	271(90.9)	160(93.6)	111(87.4)	
M1	27(9.1)	11(6.4)	16(12.6)	
**N(%)**				0.915
N0	38(12.8)	21(12.3)	17(13.4)	
N1	260(87.2)	150(87.7)	110(86.6)	

Samples with unclear clinical information were excluded (n = 298).

Values in parentheses are percentages.

P < 0.05 was regarded as statistical significance (Chi-squared test).

Statistically significant values are in bold font. SD: Standard Deviation.

N: N0: Patients with no lymph node metastasis; N1: Patients with lymph node metastasis.

M: M0: Patients with no distant metastasis; M1: Patients with distant metastasis.

**Table 2.** Correlation between ACTN1 expression and clinicopathologic characteristics of gastric cancer patients (GSE84437)

N	Overall	low	high	*p*
	431	231	200	
**Age(mean(SD))**	60.02(11.58)	59.74(11.37)	60.33(11.83)	0.601
**Gender(%)**				1
Female	137(31.8)	73(31.6)	64(32.0)	
Male	294(68.2)	158(68.4)	136(68.0)	
**T(%)**				**0.002**
T1+ T2	49 (11.4)	37 (16.0)	12(6.0)	
T3+ T4	382(88.6)	194(84.0)	188(94.0)	
**N(%)**				1
N0	80(18.6)	43(18.6)	37(18.5)	
N1	351(81.4)	188(81.4)	163(81.5)	

Samples with unclear clinical information were excluded (n = 431).

Values in parentheses are percentages.

P < 0.05 was regarded as statistical significance (Chi-squared test).

Statistically significant values are in bold font. SD: Standard Deviation.

N: N0: Patients with no lymph node metastasis; N1: Patients with lymph node metastasis.

**Figure 2.**
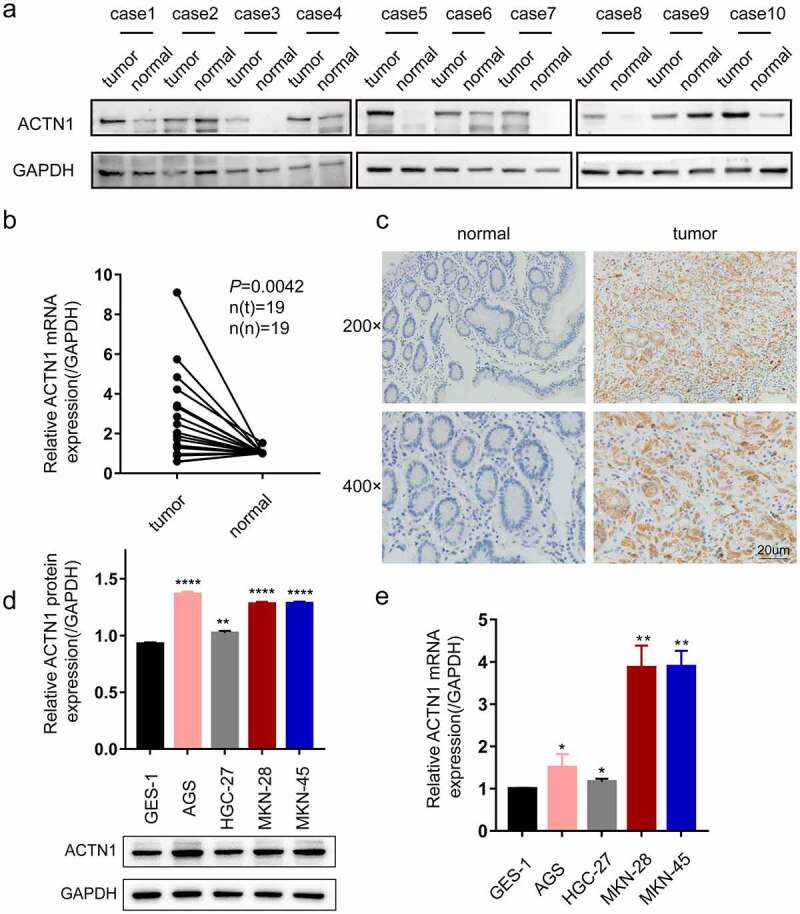
**Aberrant expression of ACTN1 on levels of GC tissue and cell line. a** Western blotting showed that ACTN1 was up-regulated in 8 of 10 pairs of GC tumor samples (tumor) relative to adjacent normal tumors (normal). **b** The mRNA level of ACTN1 between 19 pairs of gastric cancer (GC) tumors (tumor) and adjacent normal tumors (normal) by RT-qPCR. **c** The representative immunohistochemistry of ACTN1 (magnification: 200×; 400×; Scale bars 20um). **d**-and **e**. Protein (d) and mRNA (e) levels of ACTN1 between human gastric epithelial cell line (GES-1) and GC cells (HGC-27, AGS, MKN-45, and MKN-28) (P < 0.05 *, <0.01 **, <0.001 ***, <0.0001 ****; GAPDH as loading control for RT-qPCR and WB)

Subsequently, the protein level of ACTN1 was assessed by WB, which showed that 8 of the 10 pairs had increased levels of ACTN1 within GC samples relative to adjacent samples; this finding was further verified by RT-qPCR of 19 GC samples and matched non-carcinoma samples (P = 0.0042) (Figure 2a-b). Immunohistochemistry also indicated that four cases of normal tissues all showed negative expression, while 8 of 10 cases of GC tissues showed positive expression. (Figure 2c). Further, we determined the expression of ACTN1 in GC cell lines by WB and RT-qPCR. Compared with GES-1 (human gastric epithelial cell line), ACTN1 showed up-regulation within 4 GC cell types (AGS, HGC-27, MKN-28 and MKN-45) (Figure 2d-e). These results confirmed that ACTN1 was upregulated and predicted the poor prognostic outcome in GC. Additionally, ACTN1 was expressed in different levels among the four GC cell lines. At the mRNA level, MKN-28 had the highest ACTN1 expression. At the protein level, AGS showed the highest ACTN1 expression. Thus, we selected MKN-28 and AGS for the subsequent experiments.

### ACTN1 promoted the tumorigenesis and development of GC

**Figure 3.**
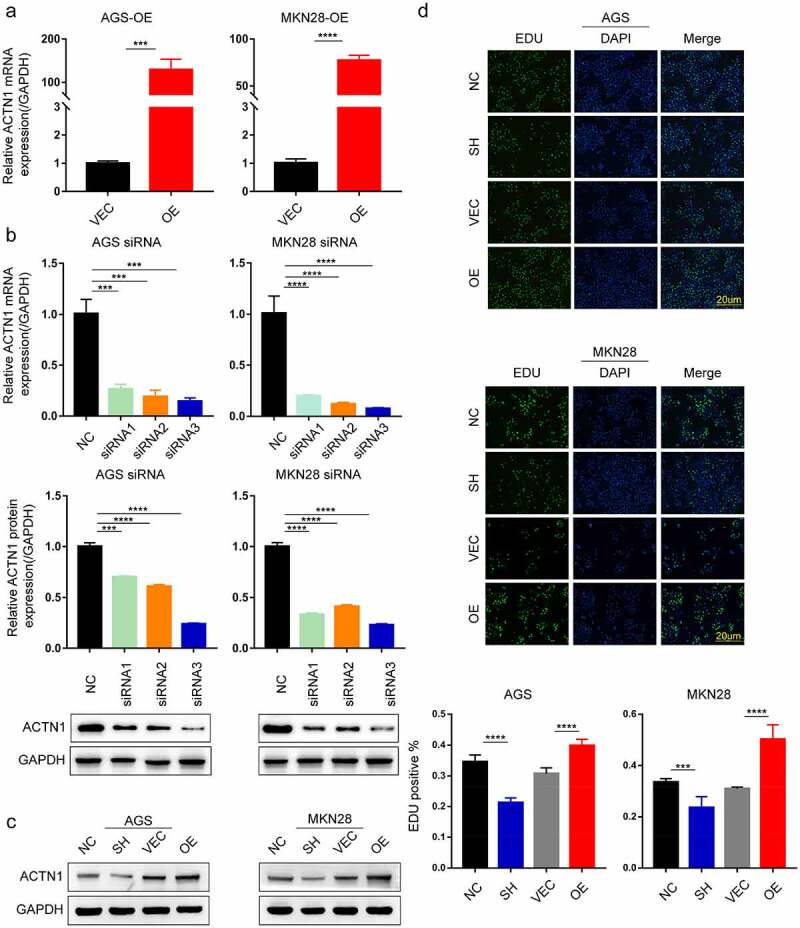
**ACTN1 promotes the proliferation of GC. a** The transfection efficiency of ACTN1 overexpression plasmid [OE] was confirmed by RT-qPCR in GC cells (with empty plasmid as negative control [VEC]). **b** RT-qPCR and WB analysis showed that siRNA3 had the highest knockdown efficiency (with siR-NC as negative control [NC]). **c** siRNA3 and ACTN1 overexpression plasmid were used for subsequent knockdown and overexpression experiments (siRNA3 [SH], siR-NC [NC], ACTN1 overexpression plasmid [OE], control plasmid [VEC]). **d** The proliferation ability of gastric cancer detected by EDU incorporation. The histogram of EDU-positive rate was quantified using the GraphPad, which showed that the overexpression of ACTN1 promoted the proliferation, whereas its knockdown reversed this effect (magnification: 100×; Scale bars 20um; P < 0.05 *, <0.01 **, <0.001 ***, <0.0001 ****)

**Figure 4.**
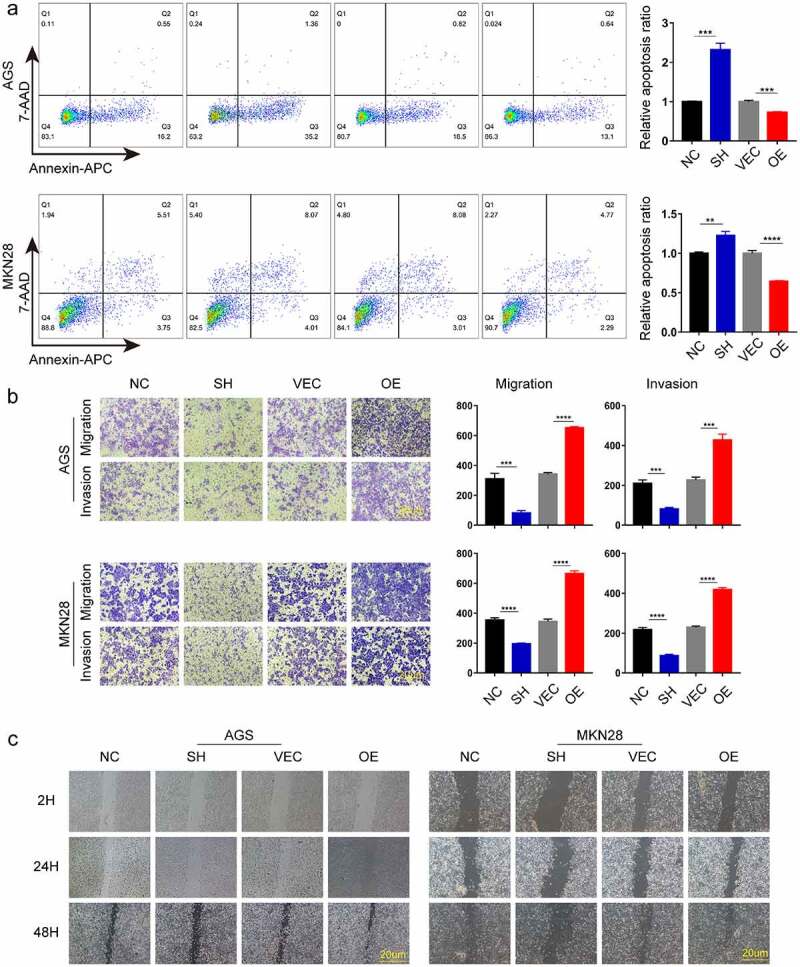
**ACTN1 promotes the invasion, migration and inhibits apoptosis of GC. a** Cell apoptosis analysis results following ACTN1 knockdown or overexpression in AGS and MKN-28 cells. **b** The invasion, and migration abilities of knock downed and overexpressed ACTN1 in gastric cancer (GC) (magnification: 100×; Scale bars 20um). **c** Wound healing assay showed that overexpressing ACTN1 promoted the migration of gastric cancer (GC), whereas its knockdown suppressed the migration of GC (magnification: 40×; Scale bars 20um) (P < 0.05 *, <0.01 **, <0.001 ***, <0.0001 ****)

To determine the effect of ACTN1 on GC, we used small interfering RNA (RNAi) and plasmid vectors to either knockdown or overexpress ACTN1 in GC cells, which was verified by WB and RT-qPCR (Figure 3a-c, Supplement Fig. S3). The results showed that ACTN1 overexpression plasmid was considerably efficient and siRNA3 had the best knockdown efficiency among the three siRNAs. Thus, the siRNA3 and overexpression plasmid were used in subsequent experiments. Then the EDU incorporation, the assay revealing the proliferation ability of cells, showed that knocking down ACTN1 significantly reduced the proliferation ability of gastric cancer cells, while the overexpression enhanced the proliferation ability (Figure 3d). Through flow cytometry analysis, we analyzed the apoptosis rate of GC and found that the apoptosis rate of ACTN1-silencing GC cells was significantly increased, while that decreased obviously after overexpressing ACTN1 (Figure 4a). Subsequently, the transwell assay showed that silencing ACTN1 significantly reduced the invasive and migratory ability of GC, while overexpression enhanced it (Figure 4b). The wound healing experiment verified the influence of ACTN1 on the migration of GC (Figure 4c). In summary, ACTN1 promoted the tumorigenesis and development of GC.

### ACTN1 regulated EMT in GC

**Figure 5.**
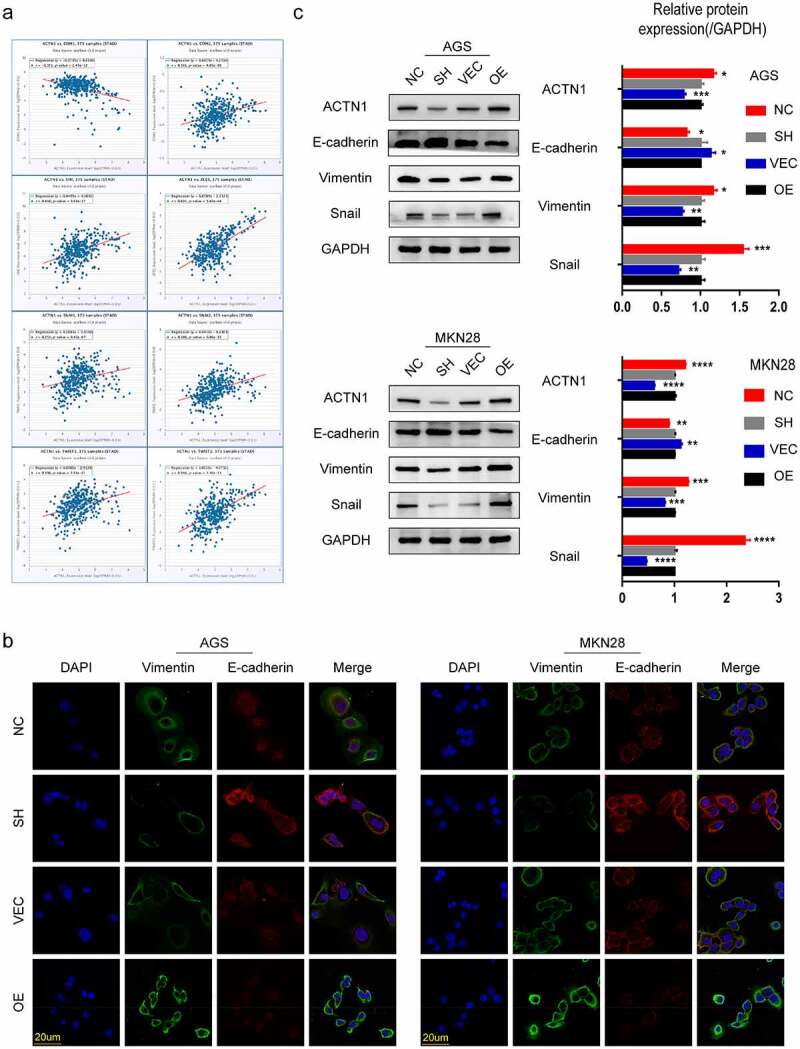
**ACTN1 regulates EMT in gastric cancer. a** Correlation analysis via bioinformatics website (The Encyclopedia of RNA Interactomes ENCORI) shows that ACTN1 is positively related to mesenchymal markers (CDH2, VIM, SNAI1, SNAI2, ZEB1, TWIST1, TWIST2) and negatively related to the epithelial marker (CDH1) (P < 0.0001). **b** Co-immunofluorescence of E-cadherin (the epithelial marker) and Vimentin (the mesenchymal marker) revealed that ACTN1 up-regulation remarkably up-regulated Vimentin whereas down-regulated E-cadherin (Vimentin stained green; E-cadherin stained red; nuclei stained blue [DAPI]). **c** Protein levels of EMT-related molecules (the epithelial marker E-cadherin; the mesenchymal marker Vimentin, and the EMT-related transcription factor Snail) between ACTN1 silenced and overexpressed groups by WB (P < 0.05 *, <0.01 **, <0.001 ***, <0.0001 ****; Scale bars: 20 μm)

EMT is a key step in tumor metastasis. To determine whether ACTN1 was involved in the EMT of GC, we initially evaluated the correlation between the expression of EMT-related molecules and ACTN1 as per ENCORI. We found that the expression of ACTN1 was positively correlated with Vimentin (VIM), Snail (Snail1), Slug (Snail2), ZEB1, TWIST1, TWIST2, and N-cadherin (CDH2), which are mesenchymal cell markers. Moreover, ACTN1 expression was negatively correlated with the epithelial marker E-cadherin (CDH1). These significant corelations indicated that aberrant ACTN1 might play a role in the EMT of GC (Figure 5a).

To confirm our speculation, we used the mesenchymal cell marker Vimentin, the epithelial marker E-cadherin, and the EMT-related transcription factor Snail to evaluate the EMT of cell lines ectopically overexpressing and under expressing ACTN1 [26,29,54,55]. As shown in Figure 5b, Vimentin and E-cadherin expression levels and locations were measured through immunofluorescence. We found that overexpression of ACTN1 had significantly increased Vimentin expression whereas decreased E-cadherin expression simultaneously. We next examined the EMT-related biomarkers using WB. The results obtained were consistent with those of immunofluorescence assay, showing that in both cell lines overexpressing ACTN1, the expression of Vimentin and Snail increased and that of E-cadherin decreased, whereas opposite results were observed in both ACTN1 silent GC cells (Figure 5c). Overall, these results indicated that ACTN1 promoted EMT in GC.

### ACTN1 promoted the expression and nuclear translocation of β-catenin

**Figure 6.**
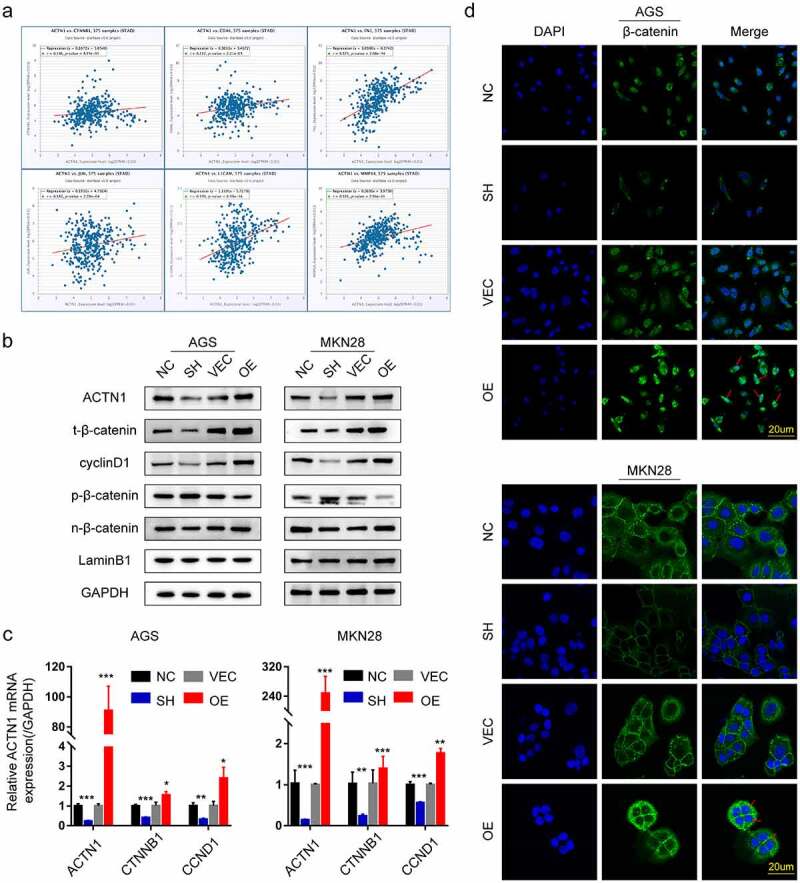
**ACTN1 promotes stability and nuclear translocation of β-catenin. a** The ENCORI shows that ACTN1 is positively related to β-catenin (CTNNB1) and its downstream target genes (CD44, FN1, C-JUN, L1CAM, MMP14) (P < 0.001). **b** Western blotting showed that the ACTN1 overexpression increased the protein levels of total β-catenin (t-β-catenin), nuclear β-catenin (n-β-catenin), and cyclin D1, whereas it inhibited the protein levels of p-β-catenin (phosphorylated at T41 and S45). The knockdown of ACTN1 produced the opposite effects (LaminB1 as loading control for nucleoprotein). **c** Transcription levels of cyclin D1 (CCND1) and β-catenin (CTNNB1) were assessed between ACTN1 silenced and overexpressed groups by RT-qPCR (P < 0.05 *, <0.01 **, <0.001 ***, <0.0001 ****). **d** Immunofluorescence showed that ACTN1 overexpression significantly up-regulated cytoplasmic β-catenin level, with a simultaneously moderate increase of β-catenin in the nucleus which is indicated by a red arrow. The knockdown of ACTN1 produced the opposite effects (Scale bars: 20 μm)

We next tried to determine the mechanism by which ACTN1 promotes the EMT of GC using the bioinformatic tools. Interestingly, the results obtained from ENCORI and GEPIA showed positive correlations of ACTN1 with β-catenin (CTNNB1) and major corresponding downstream target genes such as C-Myc, cyclinD1 (CCND1), CD44, L1CAM, C-JUN, FN1, and MMP14 (Figure 6a, Supplement Fig. S1c). Notably, the Wnt/β-catenin signal transduction pathway has been suggested to modulate tumor genesis and EMT in certain malignancies [32,33]. We speculated that ACTN1 might be related to the Wnt/β-catenin signal transduction pathway. Thereafter, we performed WB to confirm our speculations. As expected, the overexpression of ACTN1 significantly increased the protein levels of β-catenin and its downstream target gene cyclin D1, decreasing the phosphorylation level of β-catenin. Moreover, the levels of nuclear β-catenin increased significantly, whereas knockdown of ACTN1 produced the opposite effect (Figure 6b). Further, Figure 6c illustrated that overexpression of ACTN1 significantly prompted the transcription level of β-catenin and cyclinD1, while silencing ACTN1 down-regulated the transcription level β-catenin and cyclinD1. This study also measured β-catenin level and subcellular localization in both GC cell lines by immunofluorescence. Consistent with the above results, the overexpression of ACTN1 significantly increased the cytoplasmic β-catenin level; however, an inconspicuous increase was detected in the nucleus. It could be beacuse a few β-catenin molecules in the nucleus were sufficient to change the transcription level of cells that were not enough to be detected by immunofluorescence (Figure 6d) [32]. Overall, we demonstrated that ACTN1 enhanced the β-catenin stabilization and nuclear import.

### ACTN1 accelerated the tumorigenesis and regulated the EMT of GC via the AKT/GSK3β/β-catenin pathway

**Figure 7.**
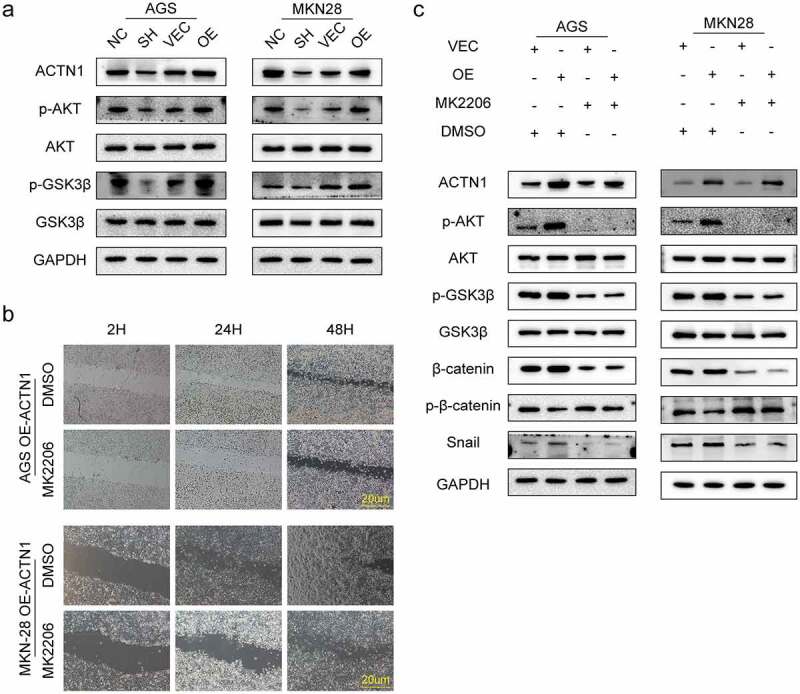
**ACTN1 promotes the tumorigenesis and development of gastric cancer through the AKT/GSK3β/β-catenin pathway and promotes EMT. a** The overexpression of ACTN1 significantly promoted the phosphorylation of AKT (Ser473) and GSK3β (Ser9), whereas its knockdown inhibited this phosphorylation. **b** The AKT inhibitor MK2206 (10 mM) significantly rescued the migration-promoting effect of overexpressed ACTN1 in gastric cancer (GC) cells (Dimethylsulfoxide [DMSO] as control; Scale bars: 20 μm). **c** Western blotting revealed that ACTN1 up-regulation enhanced p-AKT, p-GSK3β, β-catenin, and Snail expression, and inhibited the phosphorylation of β-catenin. The AKT inhibitor MK2206 (10 mM) partially rescued the effect of ACTN1

GSK3β, a member of the β-catenin destruction complex, exerts an important part in β-catenin phosphorylation. AKT-mediated phosphorylation at Ser9 inhibits the catalytic activity of GSK3β, thus accumulating β-catenin in the cytoplasm and inducing transcription of its downstream target genes. Therefore, we investigated whether the AKT/GSK3β pathway regulated β-catenin due to the deregulation of ACTN1 in GC. We assessed the total and phosphorylated AKT, and GSK3β expression in MKN-28 and AGS cell lines that overexpressed or under expressed ACTN1. As expected, the overexpression of ACTN1 significantly facilitated the phosphorylation of AKT and GSK3β. In contrast, the knockdown of ACTN1 reversed this effect (Figure 7a).

To further clarify whether the AKT/GSK3β/β-catenin pathway regulated the ACTN1 overexpression-induced migration abilities of GC, we treated GC cell lines overexpressing ACTN1 with 10 µL of MK2206 (an AKT inhibitor). Dimethyl sulfoxide (DMSO) was used as the negative control. We found that MK2206 significantly inhibited the ACTNI overexpression-induced migration abilities (Figure 7b). Western blotting results showed that MK2206 treatment of GC cells overexpressing ACTN1 significantly inhibited the GSK3β phosphorylation, β-catenin and EMT transcription inhibitor Snail expression, whereas enhanced β-catenin phosphorylation (Figure 7c). The AKT inhibitor MK2206 definitely rescued the effect of ACTN1 overexpression. Collectively, these findings indicated that ACTN1 promoted the carcinogenesis of GC cells and EMT, which executed via the activation of AKT/GSK3β/β-catenin signaling.

## Discussion

Gastric cancer is still a major health issue worldwide due to the lack of sensitive diagnostic indicators and efficient therapeutic strategies [1–3]. In this study, we aimed to elucidate the mechanism of tumorigenesis of GC and determine the potential targets of therapy. The differentially expressed genes (DEGs) between tumor tissues and tumor-adjacent tissues were identified by high-throughput sequencing for providing clues to determine the mechanisms. After collecting evidence using different approaches, we found that ACTN1 was overexpressed in tumor tissues and associated with significantly poor prognostic outcomes, which indicated that ACTN1 might act as an oncogene in GC.

ACTN1, an actin cross-linking protein, is widely distributed in several cells and tissues, associating with diverse signaling mechanisms such as phosphatidylinositol 3-kinase (PI3K), ERK, protein kinase N (PKN), and MEKK1, implying crucial roles of ACTN1 [56–59]. Aberrant expression of ACTN1 has been discovered among certain tumors, and functions as an oncogene. In hepatocellular carcinoma (HCC), ACTN1 has been demonstrated to promote tumorigenesis of HCC by inhibiting Hippo signaling [18]. Simultaneously, the malicious roles of ACTN1 in breast cancer have also been proved. Vallenius et al. found that aberrant ACTN1 destabilized E-cadherin-based adhesions to prompt the migration of breast cancer cells [16]. Ezharul Hoque Chowdhury et al. successfully inhibited the proliferation of breast cancer cells utilizing pH sensitive nanoparticle-mediated delivery of ACTN1-siRNA [22]. Gao et al. found that ACTN1 up-expression in cancer-associated fibroblasts (CAFs) promoted breast tumor metastasis through activating FAK/Src/JAK2/STAT3 signaling, which inhibited by Oroxylin A [17]. Of greater interest is that Basson et al. demonstrated ACTN1 acted as a mediator in converting physical forces into intracellular signals, which in turn derived the proliferation, motility, as well as formation and turnover of focal adhesion in colon cancer. This may provide theoretical supports for a new method based on ACTN1 to prevent tumor migration caused iatrogenically by surgical manipulation [23]. These studies testified that ACTN1 occupied a vital part in tumor progression through diverse aspects, providing potential gene therapy targets. However, the role of ACTN1 has not yet been reported in gastric cancer. In our study, we for the first time demonstrated that ACTN1, acting as an oncogene, prompts tumorigenesis and EMT of gastric cancer, and subsequently explored the underlying mechanism.

The high migration ability of cancer cells contributes to poor prognosis, with EMT playing a pivotal function in malignant invasion and metastasis [24,25,28–30]. In the course of EMT, destabilizing of E-cadherin-based adhesion results in the breakdown of cell-cell adhesion and prompts the migration of tumor cells. Reports have indicated that aberrant ACTN1 expression was responsible for the EMT in several cancers, such as the oral squamous cell carcinoma [16,20]. We investigated whether ACTN1 regulated the EMT in GC by evaluating biomarkers of EMT in different cell lines ectopically overexpressing and underexpressing ACTN1. Consistent with these studies, we found that the upregulation of ACTN1 had significantly increased Snail and Vimentin expression whereas decreased E-cadherin expression, which indicated that ACTN1 facilitated the EMT of gastric cancer.

The literature reports that numerous signaling pathways are associated with aberrant EMT of cancers, such as AKT, NF-kB and Wnt/β-catenin [31,35,36,39]. AKT, a serine/threonine kinase, plays an essential role in the PI3K/AKT signaling cascade [39]. Abnormal phosphorylation of AKT is related to poor malignancy and prognosis of cancer . AKT can phosphorylate GSK3β at Ser9 to inhibit its catalytic activity, which enhances Snail stability, and promote EMT of tumors by down-regulating its target gene E-cadherin [38,41,44,55]. The abnormal AKT/GSK3β/Snail signaling has already been identified to relate with the progression of tumors in several cancers, including GC [60,61]. Likewise, GSK3β act as a negative regulator within the Wnt/β-catenin signaling by degrading β-catenin [30,38]. When GSK3β is inhibited by AKT, free β-catenin accumulate and translocate to the nucleus, ultimately activating downstream target genes such as cyclin D1, C-Myc, CD44, FN1, C-JUN, Slug, L1CAM, and MMP14; thus contributing to tumorigenesis and EMT of cancer [37]. Studies have demonstrared that the aberrantly activated AKT/GSK3β/β-Catenin signal pathway plays a crucial part in malignant progress [38,39,42,62]. After a detail investigation of the mechanism, we found that ACTN1 upregulation enhanced the phosphorylation of GSK3β and AKT, as well as, Snail expression levels in gastric cancer. Moreover, ACTN1 overexpression led to the nuclear import β-catenin and upregulated the target gene cyclinD1, whereas its silencing produced the opposite effect. Rescue experiences using the AKT inhibitor MK2206 tested our conclusion consistently. As a result, we initially verified that ACTN1 was upregulated in GC and facilitated tumorigenesis and EMT of GC via the AKT/GSK3β/β-Catenin pathway. Further, our research also has some limitations, such as the lack of in vivo experiments and limited sample size.

It is worth noting that our research still needs to be in-depth. We have not studied how the abnormal expression of ACTN1 leads to the phosphorylation of AKT. And based on the multiple positioning and relevant researches of ACTN1, we have reason to suspect that the AKT signaling may not be the unique mechanism of ACTN1 functions in GC. This study showed that ACTN1 simultaneously impacted the expression of β-catenin at the levels of mRNA and protein both, which means ACTN1 impacts the expression of β-catenin via a strong synergetic effect. The role of ACTN1 in gastric cancer still needs to be further developed.

## Conclusion

In summary, ACTN1 participated in the tumorigenesis and EMT of GC via the AKT/GSK3β/β-Catenin pathway and was correlated to poor prognosis, and could be a promising candidate for GC treatment.

## Data Availability

The datasets and experimental data used and/or analyzed in this study are available from the corresponding author on reasonable request.

## Highlights

1. The role of ACTN1 in the occurrence and development of gastric cancer was first identified.

2. Mechanistic studies revealed that ACTN1 regulates the epithelial-mesenchymal transition (EMT) and tumorigenesis of gastric cancer via the AKT/GSK3β/β-catenin pathway.

3. ACTN1 could be a promising candidate for GC treatment.

## Abbreviations

α-Actinin1(ACTN1); Gastric Cancer (GC); Gene Expression Omnibus (GEO); The Cancer Genome Atlas (TCGA); Western Blotting (WB); Real-Time Quantitative polymerase chain reaction (RT-qPCR); Epithelial-Mesenchymal Transition (EMT); Breast Cancer (BC); Phenylmethylsulfonyl fluoride (PMSF); Tris Buffered Saline Tween (TBST); Glyceraldehyde-3-phosphate dehydrogenase (GAPDH); Phosphate buffered saline (PBS); Standard Deviation (SD); Dimethyl sulfoxide (DMSO); The differentially expressed genes (DEGs); Phosphatidylinositol 3-kinase (PI3K).
